# Plasma microRNA profiles: identification of miR-1229-3p as a novel chemoresistant and prognostic biomarker in gastric cancer

**DOI:** 10.1038/s41598-020-59939-8

**Published:** 2020-02-21

**Authors:** Keiji Nishibeppu, Shuhei Komatsu, Taisuke Imamura, Jun Kiuchi, Takuma Kishimoto, Tomohiro Arita, Toshiyuki Kosuga, Hirotaka Konishi, Takeshi Kubota, Atsushi Shiozaki, Hitoshi Fujiwara, Kazuma Okamoto, Eigo Otsuji

**Affiliations:** Division of Digestive Surgery, Department of Surgery, Kyoto Prefectural University of Medicine, 465 Kajii-cho, Kawaramachihirokoji, Kamigyo-ku Kyoto, 602-8566 Japan

**Keywords:** Chemotherapy, Gastric cancer

## Abstract

This study aimed to explore novel microRNAs in plasma for predicting chemoresistance in adjuvant chemotherapy for patients with gastric cancer (GC). We used the Toray 3D-Gene microRNA array-based approach to compare preoperative plasma microRNA levels between GC patients with and without recurrences after curative gastrectomy. All patients underwent adjuvant chemotherapy with S-1, an oral fluoropyrimidine. Of 2566 candidates, six candidate microRNAs (miR-1229-3p, 1249-5p, 762, 711, 1268a and 1260b), which were highly expressed in the preoperative plasma of patients with subsequent recurrences, were selected. In a large-scale validation analysis by quantitative RT-PCR, we focused on high plasma levels of miR-1229-3p, which was an independent poor prognostic factor for recurrence free survival (*P* = 0.009, HR = 3.71). Overexpression of miR-1229-3p in GC cells induced significant chemoresistance to 5-fluorouracil (5-FU), up-regulation of thymidylate synthase (TS) and dihydroprimidine dehydrogenase (DPD) and down-regulation of SLC22A7 both *in vitro* and *in vivo*. Intraperitoneal injection of miR-1229-3p in mice induced significant chemoresistance to 5-FU, accompanied by high levels of miR-1229-3p in plasma and tumor tissue. These findings suggest that plasma miR-1229-3p might be a clinically useful biomarker for predicting chemoresistance to S-1 and selecting other or combined intensive chemotherapy regimens in GC patients.

## Introduction

Gastric cancer (GC) is the third leading cause of cancer-related death both in Japan and worldwide. Although the surgical techniques, perioperative chemotherapy regimens and perioperative management have greatly improved, GC remains one of the most common cancer types and constitutes a global health problem^1,2^. Curative gastrectomy with lymphadenectomy is recognized as an opportunity for macroscopic tumor clearance and a cure for GC. However, the effect of surgical resection is restricted to local control of the primary tumor^3,4^; thereby, it cannot prevent recurrence due to micrometastasis. Therefore, perioperative chemotherapy has been recommended to achieve microscopic tumor clearance in advanced GC. In Japan, an adjuvant chemotherapy regimen with S-1, an oral fluoropyrimidine, following curative gastrectomy for Stage II or III GC is recommended as a standard treatment to improve the survival rate of GC patients, based on the results of the ACTS-GC (Adjuvant Chemotherapy Trial of TS-1 for Gastric Cancer)^5,6^. However, some GC patients after curative gastrectomy followed by adjuvant S-1 chemotherapy had recurrences^7^. Nevertheless, no companion diagnostic marker has been detected for predicting chemoresistance to 5-fluorouracil (5-FU) or the oral modulated 5-FU prodrug S-1 in GC.

MicroRNAs (miRNAs), which are small noncoding RNAs, modulate the translation of specific protein-coding genes. miRNA was discovered in 1993, and a lot of studies have identified alterations in miRNA expression that contribute to the progression of various diseases, including the progression and development of several cancer types^8–11^. In recent decades, several studies have demonstrated that miRNAs are detectable in plasma/serum and remain in a remarkably stable form. Plasma/serum miRNAs are resistant to endogenous ribonuclease activity because they bind to certain proteins such as Argonaute 2 protein and high-density lipoproteins or are packaged by some kind of secretory vesicle, including apoptotic bodies and exosomes in plasma/serum^12,13^. Furthermore, some extracellular miRNAs are present not only through cell lysis but also through active secretion and function as intercellular transmitters. Thus, multiple blood-based miRNAs have been identified for cancer detection, monitoring of tumor dynamics and prediction of prognosis and chemoresistance.

In recent years, some researchers have demonstrated that miRNAs in plasma/serum of GC patients are useful in detecting chemosensitivity^14–18^. The relationship between plasma miRNA levels in GC patients and chemotherapy efficacy was investigated^15,16^. However, no report has elucidated the molecular mechanism of chemoresistance or comprehensively analyzed the association with blood-based miRNAs. These findings prompted us to find more novel plasma miRNA candidates that have a secure chemoresistant function in *in vitro* and *in vivo* analyses as well as human blood-based analyses, using a comprehensive miRNA array-based approach. In this study, we wished to find novel plasma miRNA candidates for predicting chemoresistance to 5-FU, using a genome-wide miRNA array-based approach. We selected six highly expressed oncogenic miRNA candidates (miR-1229-3p, 1249-5p, 762, 711, 1268a and 1260b) through a plasma miRNA array-based approach and compared the plasma levels of each miRNA between GC patients with and without recurrences after gastrectomy. All GC patients underwent curative gastrectomy and adjuvant chemotherapy with S-1. We then validated that the plasma level of miR-1229-3p was higher in GC patients with recurrences in large-scale analyses, demonstrating its prognostic and clinical value. We finally confirmed that miR-1229-3p plasma levels are related to chemoresistance in *in vitro* and *in vivo* analyses. Our results provide evidence that the plasma level of miR-1229-3p can contribute to clinical decision-making for chemotherapy in GC patients.

## Results

### Study design to find novel plasma miRNA biomarkers for chemoresistance in GC

**Figure 1:**
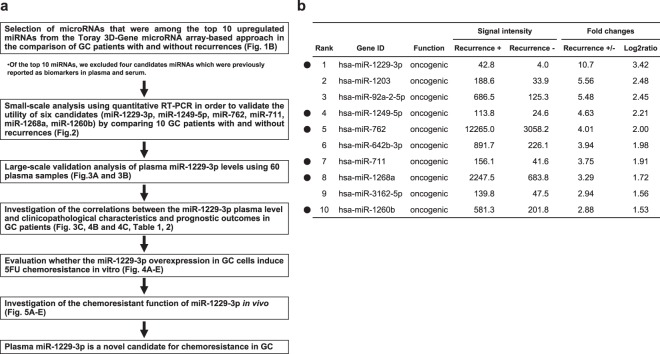
Study design and selection of plasma miRNA candidates. (**a**) Study design to find novel plasma miRNA biomarkers for chemoresistance to 5-FU in GC. (**b**) Selection of plasma miRNA candidates from a comprehensive miRNA array-based approach. Using a miRNA array-based approach, we found increases in plasma miRNAs by comparing the plasma levels of each miRNA between GC patients with and without recurrences.

We designed this study as follows: (1) Selection of appropriate miRNA candidates (miR-1229-3p, 1249-5p, 762, 711, 1268a and 1260b) based on the comparison of plasma expression level in GC patients with and without recurrence using the Toray 3D-Gene miRNA array-based approach; (2) Small-scale analysis of plasma samples using qRT-PCR to validate the usefulness of the selected miRNA candidates; (3) Large-scale analysis to validate the plasma level of miR-1229-3p; (4) Investigation of the correlations between plasma miR-1229-3p level and clinicopathological factors and prognostic outcomes in GC patients; (5) Evaluation of whether the miR-1229-3p overexpression in GC cells induced chemoresistance to 5-FU *in vitro*; and (6) Investigation of the chemoresistant function of miR-1229-3p *in vivo* (Fig. 1a).

### Selection of plasma miRNA candidates from the comprehensive miRNA array-based approach

The miRNA array based approach was used to select miRNA candidates for cancer detection based on comparison of plasma expression level in GC patients with and without recurrence. Of the 2566 candidates analyzed, the expression levels of 106 plasma miRNAs were more than two-fold higher in GC patients with recurrences than those without. To identify more sensitive biomarkers, we focused on miRNAs with top 10 expression levels in the plasma of GC patients with recurrences (Fig. 1b). Of these 10 miRNAs, we selected 6 miRNAs, miR-1229-3p, 1249-5p, 711, 762, 1268a and 1260b, which were not previously reported as tumor suppressive miRNAs and biomarkers in plasma and serum.

### Small-scale analysis of plasma levels of 6 miRNAs in GC patients with and without recurrences

**Figure 2:**
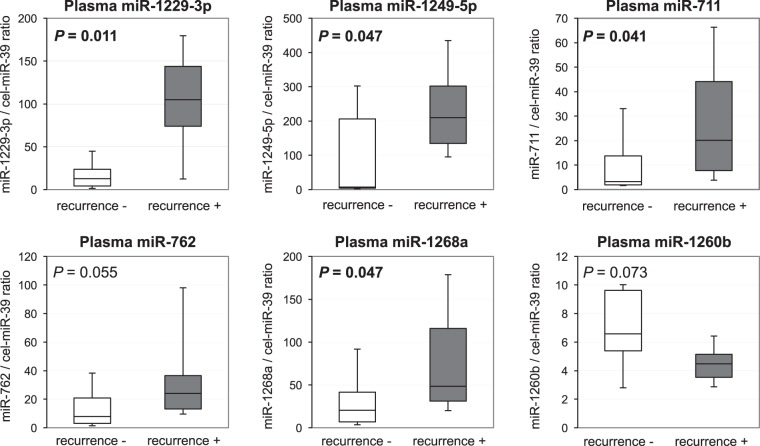
Small-scale analysis comparing plasma levels of six miRNAs between GC patients with recurrences and 10 GC patients without recurrences. Plasma levels of the selected six miRNAs in 10 GC patients with recurrences and 10 GC patients without recurrences were analyzed by qRT-PCR. The expression level of each miRNA was normalized to that of cel-miR-39 as described in Materials and Methods.

Next, we examined the expression levels of the selected six plasma miRNAs in 10 GC patients with recurrences and 10 GC patients without recurrences by qRT-PCR using small-scale analysis. As shown in the results of the miRNA array-based approach, the plasma level of miR-1229-3p (*P* = 0.011) was confirmed to be the most significant, and miR-1249-5p (*P* = 0.047), miR-711 (*P* = 0.041), miR-762 (*P* = 0.055) and miR-1268a (*P* = 0.047) tended to be higher in the plasma of GC patients with recurrences than that of GC patients without recurrences, although plasma miR-1260b (*P* = 0.073) tended to be lower in GC patients with recurrences (Fig. 2). Therefore, we selected miR-1229-3p for further analyses.

### Large-scale analysis of the plasma level of miR-1229-3p in GC patients

**Figure 3:**
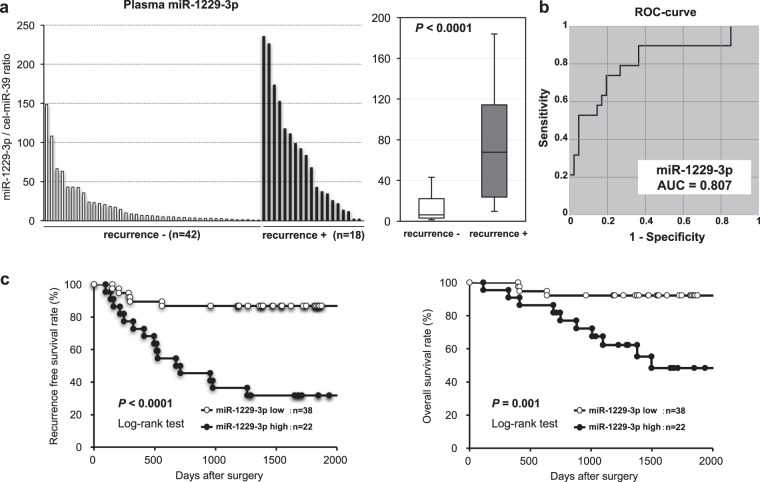
Large-scale analysis of the miR-1229-3p plasma level in GC patients with recurrences and without recurrences. (**a**) We observed that the plasma level of miR-1229-3p was significantly higher in GC patients with recurrences than in those without recurrences (*P* < 0.0001). A waterfall plot demonstrated a similar result (*P* < 0.0001). (**b**) Receiver-operating characteristic (ROC) curves and area under the ROC curve (AUC) values were used to assess the feasibility of using plasma miRNA levels as a diagnostic tool for detecting recurrences. We calculated the AUC value to be 0.807. The optimal relative expression cut-off point was indicated to be 25.8, with a sensitivity of 73.7% and a specificity of 80.5%. (**c**) A high level of plasma miR-1229-3p was significantly associated with poor prognosis.

We next validated the observations in a large-scale setting. A total of 60 plasma samples from GC patients were investigated. Plasma miR-1229-3p was detectable in all samples from 18 GC patients with recurrences and 42 GC patients without recurrences. We confirmed that the level of plasma miR-1229-3p was significantly higher in GC patients with recurrences than in GC patients without recurrences (*P* < 0.0001). A waterfall plot demonstrated a similar result (Fig. 3a). Previous reports have revealed that peripheral blood cells may release several plasma or serum miRNAs^19^. In order to exclude the possibility that miRNAs were derived from peripheral blood cells, we examined the correlation between the expression level of plasma miR-1229-3p and peripheral blood cells; no significant correlations were observed (Supplementary Fig. S1). Moreover, to evaluate the diagnostic ability of plasma miR-1229-3p and to identify the optimal cut-off value that could detect recurrences, we performed an ROC analysis (Fig. 3b). The ROC curve was formed by plotting the sensitivity against the false positive rate (1 − specificity) at various threshold settings. We utilized the AUC value and the Youden index^20^ and found that the AUC value was 0.807. The relatively optimal expression cut-off value was indicated to be 25.8, with a sensitivity of 73.7% and a specificity of 80.5%. Our results provided indication that the plasma miR-1229-3p level could be used to detect recurrences in GC patients who underwent adjuvant chemotherapy with S-1.

### Correlation between the level of plasma miR-1229-3p and clinicopathological factors in GC patients

We analyzed whether the expression level of miR-1229-3p in plasma and confirmed that clinicopathological factors were correlated in GC patients. In all 60 GC patients, advanced T-stage was the only factor correlated with a high level of plasma miR-1229-3p (*P* = 0.005; Table 1). We divided the patients into two groups and performed the analysis using the relatively optimal expression of miR-1229-3p, 25.8, in all GC patients as a cut-off. GC patients with a high level of plasma miR-1229-3p more frequently developed recurrences (*P* < 0.001), particularly peritoneal metastases (*P* < 0.001; Supplementary Table S1).

**Table 1:** Association between plasma miR-1229-3p level and clinicopathological characteristics in patients with GC.

Variables		Plasma miR-1229-3p					
		n	High		Low		*P*-value^a^
Total		60	22		38		
Gender	Male	39	14	(64%)	25	(66%)	0.866
	Female	21	8	(36%)	13	(34%)	
Age	<65	26	7	(32%)	19	(50%)	0.171
	≥65	34	15	(68%)	19	(50%)	
Tumor major axis (mm)	<50	29	7	(32%)	22	(58%)	0.064
	≥50	31	15	(68%)	16	(42%)	
T-stage	T1/T2/T3	44	11	(50%)	33	(87%)	**0.005**
	T4	16	11	(50%)	5	(13%)	
N-stage	N0/N1/ N2	47	16	(73%)	31	(82%)	0.423
	N3	13	6	(27%)	7	(18%)	
pStage	II	40	12	(55%)	28	(74%)	0.132
	III	20	10	(45%)	10	(26%)	
Histopathological type	Differentiated	21	7	(32%)	14	(37%)	0.693
	Undifferentiated	39	15	(68%)	24	(63%)	
Lymphatic invasion	Negative	14	4	(18%)	10	(26%)	0.542
	Positive	46	18	(8%)	28	(74%)	
Venous invasion	Negative	27	8	(36%)	19	(50%)	0.304
	Positive	33	14	(64%)	19	(50%)	
Recurrence	Absent	42	8	(36%)	34	(89%)	**<0.001**
	Present	18	14	(64%)	4	(11%)	

Association between plasma miR-1229-3p level and clinicopathological characteristics in patients with GC. ^a^Chi-square or Fisher tests. NOTE: significant values are in bold.

### Potential utility of plasma miR-1229-3p level as a prognostic biomarker in the plasma of GC patients

Furthermore, we have shown in prognostic analysis that a high level of plasma miR-1229-3p was significantly associated with a worse recurrence free survival (*P* < 0.0001) and overall survival (*P* = 0.001) in GC patients (Fig. 3c). We also performed a multivariate analysis for recurrence free survival rate in GC patients after curative gastrectomy (Table 2). Univariate analysis revealed that T-stage, N-stage, tumor major axis and a high plasma miR-1229-3p level were significant prognostic factors. Moreover, a multivariate analysis using the Cox proportional hazard model revealed that a high miR-1229-3p plasma level was an independent poor prognostic factor of GC patients (*P* = 0.009, hazard ratio: 3.71 (95% CI: 1.38–11.2)).

**Table 2:** Univariate and multivariate analyses of GC patient survival following gastrectomy using the Cox proportional hazards model.

	Variable	Univariate^a^	Multivariate^b^		
		*P*-value	HR^c^	95% CI^d^	*P*-value
Gender	Female *vs*. Male	0.308			
Age	≥65 *vs*. <65	0.225			
T-stage	T4 *vs*. T1/T2/T3	**<0.001**	2.41	0.92–6.49	0.072
N-stage	N3 *vs*. N0/N1/N2	**0.033**	1.77	0.68–4.28	0.229
Tumor major axis (mm)	≥50 *vs*. <50	**0.007**	2.24	0.84–7.06	0.110
Histopathological type	Undifferentiated *vs*. Differentiated	0.222			
miR-1229–3p	High *vs*. Low	**<0.001**	3.71	1.38–11.2	0.009

Univariate and multivariate analyses of GC patient survival using the Cox proportional hazards model. ^a^Univariate survival analysis was performed using the Kaplan–Meier method; the significance was determined by log-rank test. ^b^Multivariate survival analysis was performed using the Cox proportional hazards model. ^c^HR: Hazard ratio ^d^CI: Confidence interval. NOTE: significant values are in bold.

### Investigation of the miR-1229-3p function in GC cells

We investigated whether miR-1229-3p had an oncogenic function to 5-FU in GC cells *in vitro*. MiR-1229-3p mimics were transfected into HGC27 cells. Overexpression of miR-1229-3p induced cell migration and invasion. (Supplementary Fig. S2).

### Investigation of the miR-1229-3p chemoresistant function in GC cells

**Figure 4:**
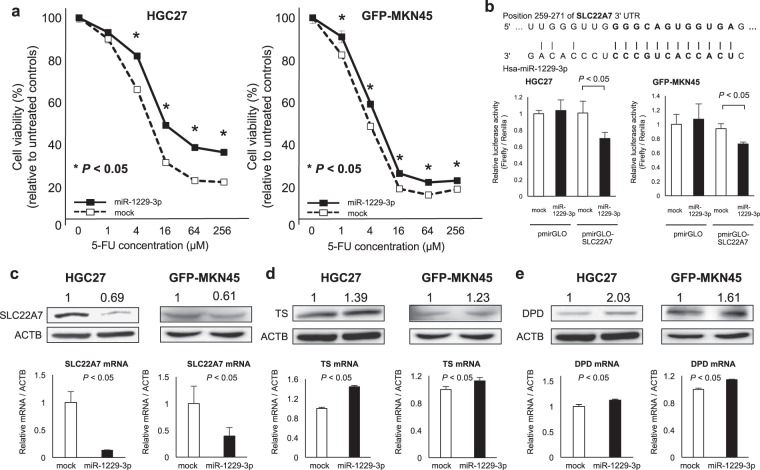
Investigation of the chemoresistance function of miR-1229-3p in GC cells. (**a**) Overexpression of miR-1229-3p significantly induced the chemoresistance to 5-FU in HGC27 and GFP-MKN45 cells. (**b**) SLC22A7 as a novel target gene of miR-1229-3p in GC cells. An *in silico* search (http://www.targetscan.org/) identified SLC22A7 as a novel target gene of miR-1229-3p in GC. The seed regions of the miR-1229-3p and complementary *SLC22A7* 3′UTR sequences are presented in this figure. Overexpression of miR-1229-3p significantly reduced luciferase activity of the pmirGLO-SLC22A7 construct with cloned 3′UTR sequences of SLC22A7 in HGC27 and GFP-MKN45 cells. (**c**) MiR-1229-3p overexpression inhibited SLC22A7 mRNA and protein production. (**d,e**) TS and DPD of mRNA and protein levels were increased at 72 h after miR-1229-3p mimics transfection.

Next, we investigated whether miR-1229-3p had a chemoresistant function to 5-FU in GC cells *in vitro*. MiR-1229-3p mimics were transfected into HGC27 (mutant *TP53*) and GFP-MKN45 (wild-type *TP53*). After the overexpression of miR-1229-3p was confirmed, the transfected HGC27 and GFP-MKN45 cells were treated with increasing concentrations of 5-FU, and cell viability was measured using the WST-8 assay. The viability of mock HGC27 and GFP-MKN45 cells, which transfected with only Lipofectamine RNAiMAX, was obviously inhibited by 5-FU, whereas the 5-FU inhibitory effect was significantly reduced in miR-1229-3p-transfected HGC27 and GFP-MKN45 cells (Fig. 4a).

Moreover, we investigated the chemoresistant mechanisms of miR-1229-3p. To investigate whether miR-1229-3p directly regulates novel target genes, we focused on the SLC22A7 gene, which has been reported to be a transporter of 5-FU in cancer cells^21^ and a putative target using TargetScan (http://www.targetscan.org/). The miR-1229-3p seed regions and complementary *SLC22A7* 3′UTR sequences are presented in Fig. 4b. Then we used the Dual-GLO Luciferase assay system to determine whether the reduction in SLC22A7 levels by overexpression of miR-1229-3p through the 3′UTR of the targeted mRNA. Overexpression of miR-1229-3p significantly reduced luciferase activity of the pmirGLO-construct with cloned 3′UTR sequences of SLC22A7 compared with co-transfected mimic control (Fig. 4b). The overexpression of miR-1229-3p inhibited the production of SLC22A7 mRNA and protein (Fig. 4c). We also confirmed that mRNA and protein levels of both thymidylate synthase (TS) and dihydroprimidine dehydrogenase (DPD), which were reported as an inhibitor of 5FU^22–25^, were increased at 72 h after miR-1229-3p mimics transfection (Fig. 4d,e). These findings suggested that SLC22A7 is a candidate for direct target of miR-1229-3p. Thus, miR-1229-3p had a chemoresistant function to 5-FU in GC cells *in vitro*.

### Overexpression of miR-1229-3p induced chemoresistance to 5-FU *in vivo*

**Figure 5:**
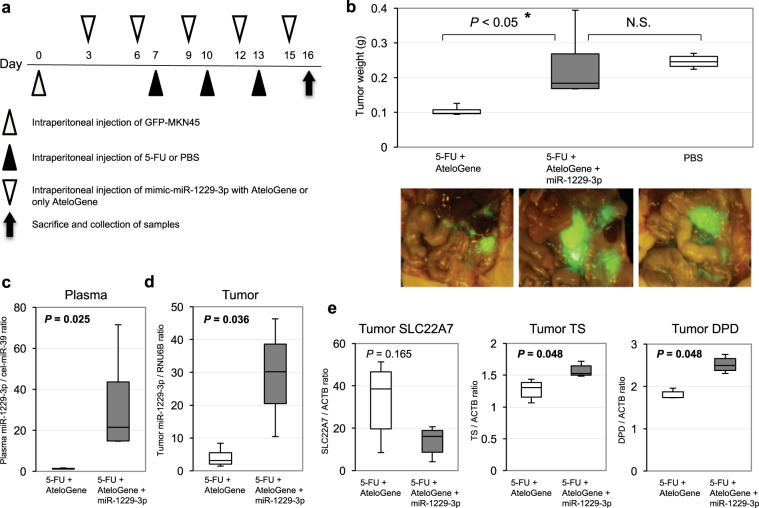
Overexpression of miR-1229-3p induced chemoresistance to 5-FU *in vivo*. (**a**) Investigation into whether miR-1229-3p could induce chemoresistance *in vivo*. To evaluate the chemoresistant function of miR-1229-3p, intraperitoneal injection of miR-1229-3p with AteloGene or only AteloGene was repeated every 3 days for 2 weeks. Furthermore, 5-FU or PBS was injected into the intraperitoneal cavity on the day after the injection of the AteloGene with or without miR-1229-3p mimics. In addition to these two treatment groups, the result of non-treatment group was checked. (**b**) The miR-1229-3p mimic significantly induced chemoresistance to 5-FU compared with the control. Error bars indicate s.e.m; n = 4 mice per group. (**c,d**) Plasma and tumor miR-1229-3p levels were significantly higher in mice with miR-1229-3p mimics than only AteloGene. Error bars indicate s.e.m; n = 4 mice per group. (**e**) Tumor SLC22A7 level was lower in mice with miR-1229-3p than with only AteloGene. Moreover, tumor TS and DPD levels were significantly higher in mice with miR-1229-3p than with only AteloGene.

Peritoneal recurrence was the most significant factors associated with high levels of miR-1229-3p (Supplementary Table S1). We also investigated the chemoresistant function and mechanisms of miR-1229-3p in an *in vivo* model using SCID mice with intraperitoneal GC tumors. Every three days after the intraperitoneal injection of GFP-MKN45 cells, the miR-1229-3p with atelocollagen or only atelocollagen was injected into the intraperitoneal cavity. Also, we injected 5-FU into the intraperitoneal cavity on the day after the injection of atelocollagen with or without miR-1229-3p mimics (Fig. 5a). In Fig. 5b, the mice treated with miR-1229-3p mimics enhanced GFP more than those without miR-1229-3p mimics. Furthermore, tumor weight was significantly higher in mice treated with miR-1229-3p mimic than those without miR-1229-3p mimics (Fig. 5b). The expression levels of plasma and tumor miR-1229-3p were significantly higher in mice treated with miR-1229-3p than in control mice treated with only atelocollagen (Fig. 5c,d). As shown by the *in vitro* analyses, we also confirmed *in vivo* that tumor SLC22A7 level was lower in mice treated with miR-1229-3p mimics than in control mice treated with only atelocollagen. Moreover, we also confirmed that tumor TS and DPD levels were significantly higher in mice treated with miR-1229-3p mimics than in control mice treated with only atelocollagen. These findings strongly indicated that overexpression of miR-1229-3p induced chemoresistance to 5-FU *in vivo* as well as *in vitro* (Fig. 5e).

## Discussion

Detection of clinically useful biomarkers for predicting chemoresistance to key anticancer drugs, such as 5-FU in GC patients, is urgently required to reduce the recurrence rate following adjuvant chemotherapy and obtain a response after recurrences during chemotherapy. In the present study, we identified a novel blood-based biomarker, miR-1229-3p, for chemoresistance to 5-FU, through genome-wide miRNA profiling of the plasma of GC patients with and without recurrences using high-resolution miRNA arrays. Moreover, we confirmed that overexpression of miR-1229-3p in GC cells induced a significant chemoresistance to 5-FU, up-regulation of TS and DPD and down-regulation of SLC22A7 in *in vitro* and *in vivo* analyses. These results provide evidence that plasma miR-1229-3p could be one of several pivotal biomarker candidates for predicting chemoresistance to 5-FU and contribute to the selection of 5-FU based chemotherapy regimens or other intensive chemotherapy regimens in GC patients.

Concerning the molecular functions of miR-1229-3p, Tan *et al*. revealed that miR-1229 was overexpressed in breast cancer, and miR-1229 inhibited the expression of GSK-3β, APC and ICAT, the vital negative regulators of the Wnt/β-catenin pathway^26^. In this study, the expression level of plasma miR-1229-3p was significantly higher in GC patients with recurrences than in those without. Also, miR-1229-3p induced chemoresistance to 5-FU in GC cells through the up-regulation of TS and DPD. TS catalyzes the reductive methylation of deoxyuridine monophosphate (dUMP) to deoxythymidine monophosphate (dTMP), with the reduced folate cofactor 5,10-methylenetetrahydrofolate (CH2THF)^27–29^. Nakamura *et al*. revealed that high expression of TS induced 5-FU resistance in GC cell lines^30^. DPD has been reported as a rate-limiting enzyme in the catabolism of 5-FU^22^. Several studies have indicated that high expression of DPD is associated with resistance to 5-FU in GC cells^22–25^. In this study, overexpression of miR-1229-3p induced the up-regulation of TS and DPD as well as chemoresistance to 5-FU *in vitro* and *in vivo*. These results suggest that miR-1229-3p is associated with multiple chemoresistant molecules to 5-FU in GC.

Moreover, we also investigated whether miR-1229-3p directly regulates novel target genes associated with chemoresistance. We focused on SLC22A7 (OAT2), which was selected as a putative target using TargetScan. SLC22A7 is reported to be a transporter of 5-FU in cancer cells. Kobayashi *et al*. revealed that high levels of SLC22A7 were related to the uptake of 5-FU^21^. Thereby, SLC22A7 is known as a predictor of the effects of various 5-FU based chemotherapies in clinical settings^31,32^. In this study, overexpression of miR-1229-3p induced the down-regulation of SLC22A7 *in vitro* and *in vivo*, so the suppression of 5-FU uptake into cancer cells would have occurred. This result may also demonstrate the chemoresistant function of miR-1229-3p to 5-FU.

Regarding the therapeutic application of our results, a combination of chemotherapy and therapeutic silencing of miR-1229-3p may suppress chemoresistance to 5-FU. Previous clinical studies have focused on the therapeutic silencing of disease-associated miRNAs using miRNA inhibitors. Miravirsen (Santaris Pharma) is one of several promising miRNA inhibitors; it can bind to miR-122 and inhibit its biogenesis. Miravirsen was developed for the treatment for hepatitis C and is currently under evaluation in clinical trials^33,34^. Therefore, if the safety of miR-1229-3p administration *in vivo* is proved, the therapeutic silencing of miR-1229-3p and removing the chemoresistance of 5-FU might also be possible. This treatment strategy is currently under evaluation, investigating the side effect to administrate the miR-1229-3p *in vivo*, and developing the new promising 5-FU drug. Also, our results were obtained from a small number of patients at a single institute. A large-scale or multicenter cohort study is necessary to confirm the utility of plasma miR-1229-3p revel in order to predict the chemoresistance of 5-FU. We will investigate these issues and report in near future.

## Methods

### Patients and samples

The study was approved by the Institutional Review Board of Kyoto Prefectural University of Medicine, and the subjects provided signed informed consent. A total of 60 plasma samples from GC patients who underwent curative gastrectomy at our institution between January 2008 and December 2013 were collected. Of all 60 samples, 18 samples were from GC patients with recurrences, and 42 samples were from GC patients without recurrences. All of the tumors were pathologically diagnosed as gastric adenocarcinoma. All patients underwent an adjuvant chemotherapy regimen with S-1 at our institute. Tumor stages were assessed according to the Union for International Cancer Control classification^35^.

Peripheral blood (7 ml) was obtained from each patient before surgery. The blood was transferred into sodium heparin tubes (BD Vacutainer, Franklin Lakes, NJ) and immediately subjected to a three-spin protocol (1500 r.p.m. for 30 min, 3000 r.p.m. for 5 min and 4500 r.p.m. for 5 min) to prevent contamination by cellular nucleic acids. Plasma was collected and then stored at −80 °C until further processing. In all cases, two pathologists agreed with the pathological observations and confirmed the diagnosis.

### RNA extraction

Total RNA was extracted from 400 μl of plasma using the mirVana PARIS Kit (Ambion, Austin, TX) and finally eluted into 100 μl of preheated (95 °C) elution solution according to the manufacturer’s protocol. A volume of 400 μl of plasma was used as the common denominator in each microarray analysis because this was the same volume used as a definite internal control in our previous studies^36–44^.

### miRNA microarray analysis

Microarray analyses of the plasma samples were performed using the 3D-Gene miRNA microarray platform (Toray Industries, Kamakura, Japan^39,41,45,46^;) The results were compared between three different GC patients with and without recurrences. Namely, each 100-μl plasma sample from three GC patients with recurrences was equally mixed to give 300 μl of plasma sample for GC patients with recurrences. Also, each 100-μl plasma sample from three GC patients without recurrences was equally mixed to give 300 μl of plasma sample for GC patients without recurrences. RNA extraction and microarray analysis were performed according to the manufacturer’s instructions, as described elsewhere^39^. Briefly, because the amount of total RNA in plasma was too small, 2 of 4 μl of extracted total RNA from the 300-μl plasma samples were used in the microarray experiments. This RNA was labelled with Hy5 using the Label IT miRNA Labeling Kit (Takara Bio, Otsu, Japan) and hybridized at 32 °C for 16 h on a 3D-Gene chip. The 3D-Gene miRNA microarray (Human_miRNA_v21, Toray Industries) can mount > 2500 miRNAs based on the Human miRNA Release 21 of MirBase (http://microrna.sanger.ac.uk/). The microarray was scanned, and the images obtained were enumerated using a 3D-GeneH Scanner 3000 (Toray Industries). The expression level of each miRNA was globally normalized using the background-subtracted signal intensity of the entire set of miRNAs in each microarray. The obtained microarray images were analyzed using GenePix Pro TM (Molecular Devices, Sunnyvale, CA).

### Quantification of miRNA by qRT-PCR

The amounts of miRNAs were quantified by quantitative RT-PCR (qRT-PCR) using the Human TaqMan MicroRNA Assay Kit (Applied Biosystems, Foster City, CA). The reverse transcription reaction was carried out with the TaqMan MicroRNA Reverse Transcription Kit (Applied Biosystems) in 5 μl of solution, containing 1.67 μl of extracted RNA, 0.05 μl of 100 mM dNTPs, 0.33 μl of Multiscribe Reverse Transcriptase (50 Uµl^−1^), 0.5 μl of 10× Reverse Transcription Buffer, 0.06 μl of RNase inhibitor (20 Uµl^−1^), 1 μl of gene-specific primer (Assay ID: 241038: hsa-miR-1229-3p, Assay ID: 476548; hsa-miR-1249-5p, Assay ID: 241090; hsa-miR-711, custom order hsa-miR-762 (ggggcuggggccggggccgagc), custom order hsa-miR-1268a (cgggcguggugguggggg), Assay ID: 242525; hsa-miR-1260b, Assay ID: 002410; cel-miR-39, Assay ID: 000200; and RNU6B, Assay ID: 001093, Thermo Fisher) and 1.39 μl of nuclease-free water. To synthesize cDNA, reaction mixtures were incubated at 16 °C for 30 min, at 42 °C for 30 min and at 85 °C for 5 min, and were then held at 4 °C. Next, 0.67 μl of cDNA was amplified using 5 µl of TaqMan 2× Universal PCR Master Mix with No AmpErase UNG (Applied Biosystems), 0.5 µl of gene-specific primers/probe and 3.83 µl of nuclease-free water in a final volume of 10 µl. Quantitative PCR was run on a StepOnePlus PCR system (Applied Biosystems), and reaction mixtures were incubated at 95 °C for 10 min, followed by 40 cycles of 95 °C for 15 s and 60 °C for 1 min. Cycle threshold (Ct) values were calculated with StepOne Software v2.0 (Applied Biosystems).

As previously reported^11^, we used an approach for data normalization based upon spiking the sample with a synthetic RNA oligonucleotide, cel-miR-39, which does not exist in the human genome. *C. elegans* cel-miR-39 was purchased as a custom-made RNA oligonucleotide (Qiagen, Valencia, CA). The oligo used for spiking, as a mixture of 25 fmol of oligonucleotide in 5 μl of total volume of water, was introduced after the addition of 2X Denaturing Solution (Ambion) to the plasma or serum sample to avoid degradation by endogenous plasma RNases. As a control for each RNA sample, cel-miR-39 was used for TaqMan qRT-PCR assays (Applied Biosystems) as described earlier. We normalized the data across samples using the 2^−ΔΔCt^ method relative to cel-miR-39. In contrast, expression of miRNAs from tissue samples and cultured cells was normalized using the 2^−ΔΔCt^ method relative to U6 small nuclear RNA (RNU6B). ΔCt was calculated by subtracting the Ct values of cel-miR-39 or RNU6B from those of the miRNAs of interest. ΔΔCt was then calculated by subtracting the mean of ΔCt of the plasma of GC patients with recurrence from the ΔCt of the plasma of GC patients without recurrence. The change in gene expression was calculated with the equation 2^−ΔΔCt^^47,48^.

### Culture of GC cell lines

Gastric cancer cell lines, HGC27 (mutant *TP53*) and GFP-MKN45 (wild-type *TP53*) were purchased from RIKEN Cell Bank (Tsukuba, Japan) and cultured in Roswell Park Memorial Institute (RPMI)-1640 medium (Nacalai, Japan) or Dulbecco’s Modified Eagle Medium (DMEM; Nacalai, Japan) supplemented with 10% FBS (Corning, USA). All cell lines were cultured in 5% CO_2_ at 37 °C in a humidified chamber.

### Transfection of a GC cell line with miRNA

For the overexpression of miR-1229-3p, an miR-1229-3p mimic (Assay ID: MC13382) selected from the mirVana miRNA mimic panel (Ambion) was used to transfect the HGC27 cells at a final concentration of 12 μM using Lipofectamine RNAiMAX (Invitrogen) according to the manufacturer’s instructions. After 72 h, the overexpression of miR-1229-3p was confirmed by qRT-PCR using the Human TaqMan MicroRNA Assay Kit (Applied Biosystems).

### Cell viability assays

To assess the chemoresistance of GC cell lines to 5-FU, HGC27 and GFP-MKN45 cells that were transfected with miR-1229-3p or its control were plated onto a 24-well plate (2 × 10^4^ cells/ml) and incubated overnight under normal culture conditions. The cells were then incubated with various concentrations of 5-FU (0, 1, 4, 16, 64 or 256 μM). After 72 h, these cells were subjected to a cell viability assay based on water soluble tetrazolium salts (WST)-8. Live cells were counted using the Cell Counting Kit (Dojindo Laboratories, Kumamoto, Japan).

### Transwell migration and invasion assays

Transwell migration and invasion assays were conducted in 24-well modified Boyden chambers (Transwell chambers, BD Transduction, Franklin Lakes, NJ). The upper surface of 6.4-mm-diameter filters with 8-μm pores was precoated with (invasion assay) or without (migration assay) Matrigel (BD Transduction). The miRNA mimic transfectants (5 × 10^5^ cells per well) were transferred into the upper chamber. Following 22 h of incubation, the migrated or invasive cells on the lower surface of the filters were fixed and stained with Diff-Quik stain (Sysmex, Kobe, Japan), and stained cell nuclei were counted directly in triplicate^49^.

### Western blot analysis

Anti-ACTB antibodies were purchased from Cell Signaling Technology (Cell Signaling Technology, USA). Anti-TS and anti-DPD antibodies were purchased from Abcam. Anti-SLC22A7 antibodies were purchased from TransGenic (Kumamoto, Japan). The cells were lysed in Tris buffer (50 mmol/l, pH 7.5) containing 150 mmol/L NaCl, 1 mmol/L EDTA, 0.5% NP-40, 10% glycerol, 100 mmol/L NaF, 10 mmol/L sodium pyrophosphate, 2 mmol/L Na_2_VO_3_ and a protease inhibitor cocktail (Roche, Tokyo, Japan), and their proteins were extracted using M-PER Mammalian Protein Extraction Reagent (Thermo Scientific, USA). Twenty micro-grams of proteins per lane were loaded for electrophoresis^50^. The intensities of protein bands were quantified using ImageJ software (https://imagej.nih.gov/ij/).

### Luciferase reporter assay

Seed sequences of miR-1229-3p and pairing 3′UTR sequences of SLC22A7 were predicted by TargetScan. 3′UTR sequence was cloned downstream to firefly luciferase of pmirGLO Dual-Luciferase miRNA Target Expression Vector and verified by sequencing (Promega, Madison, WI, USA). The pmirGLO or pmirGLO-SLC22A7 3′UTR construct and the mock or mimic-miR-1229-3p were co-transfected into HGC27 cells cultured in 96-well plates using Lipofectamine 2000 (Invitrogen) according to the manufacturer’s protocol. Forty-eight hours after transfection, firefly and Renilla luciferase activities were measured using a Dual-GLO Luciferase Reporter Assay System (Promega). Firefly luciferase was normalized to Renilla luciferase activity. The mean of luciferase activity of Firefly/Renilla ratio in HGC27 and GFP-MKN45 cells co-transfected with the pmirGLO constructs and mock was set to 1.

### Animal experimental protocol

For the *in vivo* model, GC cells (1 × 10^7^ GFP-MKN45, JCRB) were inoculated into the abdominal cavity of BALB/c nude mice (Shimizu Laboratory Supplies, Kyoto, Japan). Every three days after the injection of tumor cells, miR-1229-3p mimics with AteloGene Systemic use (Koken, Co., Tokyo, Japan) or only AteloGene was injected into the intraperitoneal cavity. Treatment began at 7 days after tumor cell implantation. 5-FU or PBS was injected into the intraperitoneal cavity on the day after the injection of the AteloGene with or without miR-1229-3p mimics. In addition to these two treatment groups, the result of the non-treatment group was checked. At 16 days after tumor cell implantation, the mice were sacrificed, and blood samples and cancer tissue were collected for further analysis. Fluorescence observation was performed with a stereoscopic microscope (SZX16; Olympus) equipped with a colour charge-coupled digital camera (DP73) and a mercury lamp (U-LH100HG; both Olympus). GFP fluorescence images (595–540 nm; GFPHQ cube) were acquired by excitation at 460–480 nm (GFPHQ cube; both Olympus). The animal protocol was approved by the Institutional Animal Care and Use Committee of Kyoto Prefectural University of Medicine, and all experiments were conducted strictly in accordance to the National Institute of Health Guide for Care and Use of Laboratory Animals. Four-week-old BALB/c nude mice were used in this study.

### Statistical analysis

For miRNA array-based analyses, the signal intensity ratio and log_2_ ratio of each plasma miRNA were calculated by the ratio of GC patients with recurrences to GC patients without recurrences. The Mann–Whitney U-test for unpaired data from plasma samples was performed. The Chi-square test or Fisher’s exact probability test was used to evaluate correlations between the results of plasma miRNA levels and clinicopathological factors. A *P*-value < 0.05 was considered statistically significant.

Receiver-operating characteristic (ROC) curves and the area under the ROC curve (AUC) were used to assess the feasibility of using plasma miRNA as a diagnostic tool for detecting recurrence. The ROC curve was created by plotting the sensitivity against the false positive rate (1 - specificity) at various threshold settings. The Youden index was used to determine the cutoff value for the plasma miRNAs levels^20^. For the analysis of survival rates, Kaplan–Meier survival curves were constructed for groups based on univariate predictors, and differences between the groups were analyzed with the log-rank test. Univariate and multivariate survival analyses were performed using the likelihood ratio test of the stratified Cox proportional hazards model. A *P*-value < 0.05 was considered statistically significant. This is the first report to demonstrate that miR-1229-3p, which was detected by comprehensive miRNA microarray analyses, could be a plasma biomarker for chemoresistance to 5-FU in GC. Because many problems must still be addressed before these findings can be translated into a clinically useful biomarker for GC patients, we will prospectively confirm the usefulness of plasma miR-1229-3p in more patients using digital PCR-based approaches.

### Ethics approval and informed consent

All experimental methods were carried out in accordance with relevant guidelines and regulations, such as the Declaration of Helsinki. Written informed consent was obtained from all patients to use their tissue specimens and blood samples. This study was approved by the institutional review boards of Kyoto Prefectural University of Medicine (ERB-C-319-1). The animal protocol was approved by the Institutional Animal Care and Use Committee of Kyoto Prefectural University of Medicine, and all experiments were conducted strictly in accordance to the National Institute of Health Guide for Care and Use of Laboratory Animals.

## Supplementary information

Supplementary Information.
