# Supplementary material for: Plasma microRNA profiles: identification of miR-1229-3p as a novel chemoresistant and prognostic biomarker in gastric cancer
Source: Sci Rep. 2020 Feb 21;10:3161. doi: 10.1038/s41598-020-59939-8 (PMC7035283; doi:10.1038/s41598-020-59939-8)
Supplement: Supplementary file 1 — Supplementary Information. [file 41598_2020_59939_MOESM1_ESM.docx]

**Supplementary Information**

**Title: Plasma microRNA profiles: identification of miR-1229-3p as a novel chemoresistant and prognostic biomarker in gastric cancer**

Keiji Nishibeppu, Shuhei Komatsu^*^, Taisuke Imamura, Jun Kiuchi, Takuma Kishimoto, Tomohiro Arita, Toshiyuki Kosuga, Hirotaka Konishi, Takeshi Kubota, Atsushi Shiozaki, Hitoshi Fujiwara, Kazuma Okamoto and Eigo Otsuji

Division of Digestive Surgery, Department of Surgery, Kyoto Prefectural University of Medicine, 465 Kajii-cho, Kawaramachihirokoji, Kamigyo-ku, Kyoto, 602-8566, Japan

**Corresponding author:** Shuhei Komatsu M.D., Ph.D.

Associate Professor, Division of Digestive Surgery, Department of Surgery, Kyoto Prefectural University of Medicine, 465 Kajii-cho, Kawaramachihirokoji, Kamigyo-ku, Kyoto 602-8566, Japan

Phone: +81-75-251-5527

E-mail: [skomatsu@koto.kpu-m.ac.jp](mailto:skomatsu@koto.kpu-m.ac.jp)

**Supplementary Figure 1**

The correlation between the expression level of plasma miR-1229-3p and peripheral blood cells. No significant correlations were observed between the expression level of plasma miR-1229-3p and peripheral blood cells.

**Supplementary Figure 2**

Overexpression of miR-1229-3p induced cell migration and invasion in HGC27 and GFP-MKN45 cells.

**Figure 4**

**Supplementary Table 1**

|  |  |  |  | miR-1229-3p | | | | |  |  |
| --- | --- | --- | --- | --- | --- | --- | --- | --- | --- | --- |
|  |  |  |  | High  (n=22) | |  | Low  (n=38) | |  | *P*-value^a^ |
| Recurrence |  |  |  |  |  |  |  |  |  | **<0.001** |
|  |  | Present |  | 14 | (64%) |  | 5 | (13%) |  |  |
|  |  | Absent |  | 8 | (36%) |  | 33 | (87%) |  |  |
| Lymph node |  |  |  |  |  |  |  |  |  | **0.013** |
|  |  | Present |  | 5 | (23%) |  | 1 | (3%) |  |  |
|  |  | Absent |  | 17 | (77%) |  | 37 | (97%) |  |  |
| Peritoneal |  |  |  |  |  |  |  |  |  | **<0.001** |
|  |  | Present |  | 9 | (41%) |  | 2 | (5%) |  |  |
|  |  | Absent |  | 13 | (59%) |  | 36 | (95%) |  |  |
| Hematogenous |  |  |  |  |  |  |  |  |  | 0.337 |
|  |  | Present |  | 0 | (0%) |  | 1 | (3%) |  |  |
|  |  | Absent |  | 22 | (100%) |  | 37 | (97%) |  |  |
| Local |  |  |  |  |  |  |  |  |  | 0.691 |
|  |  | Present |  | 1 | (5%) |  | 1 | (3%) |  |  |
|  |  | Absent |  | 21 | (95%) |  | 37 | (97%) |  |  |

Comparison of recurrence patterns according to whether miR-1229-3p high or low.
^a^ P-values are from x^2^ or Fisher's exact test. NOTE: Significant values are in bold.
